# Supplementary material for: Time‑resolved multi-omic analysis of paclitaxel exposure in human iPSC‑derived sensory neurons unveils mechanisms of chemotherapy‑induced peripheral neuropathy
Source: Cell Death Dis. 2026 Feb 10;17(1):211. doi: 10.1038/s41419-026-08445-2 (PMC12921266; doi:10.1038/s41419-026-08445-2)
Supplement: Supplementary file 15 — Dataset 1. [file 41419_2026_8445_MOESM15_ESM.zip › CDD_Open_Data/05_Protein/03_Evidence_Plots_Andranik/GOBP_MICROTUBULE_DEPOLYMERIZATION.pdf]

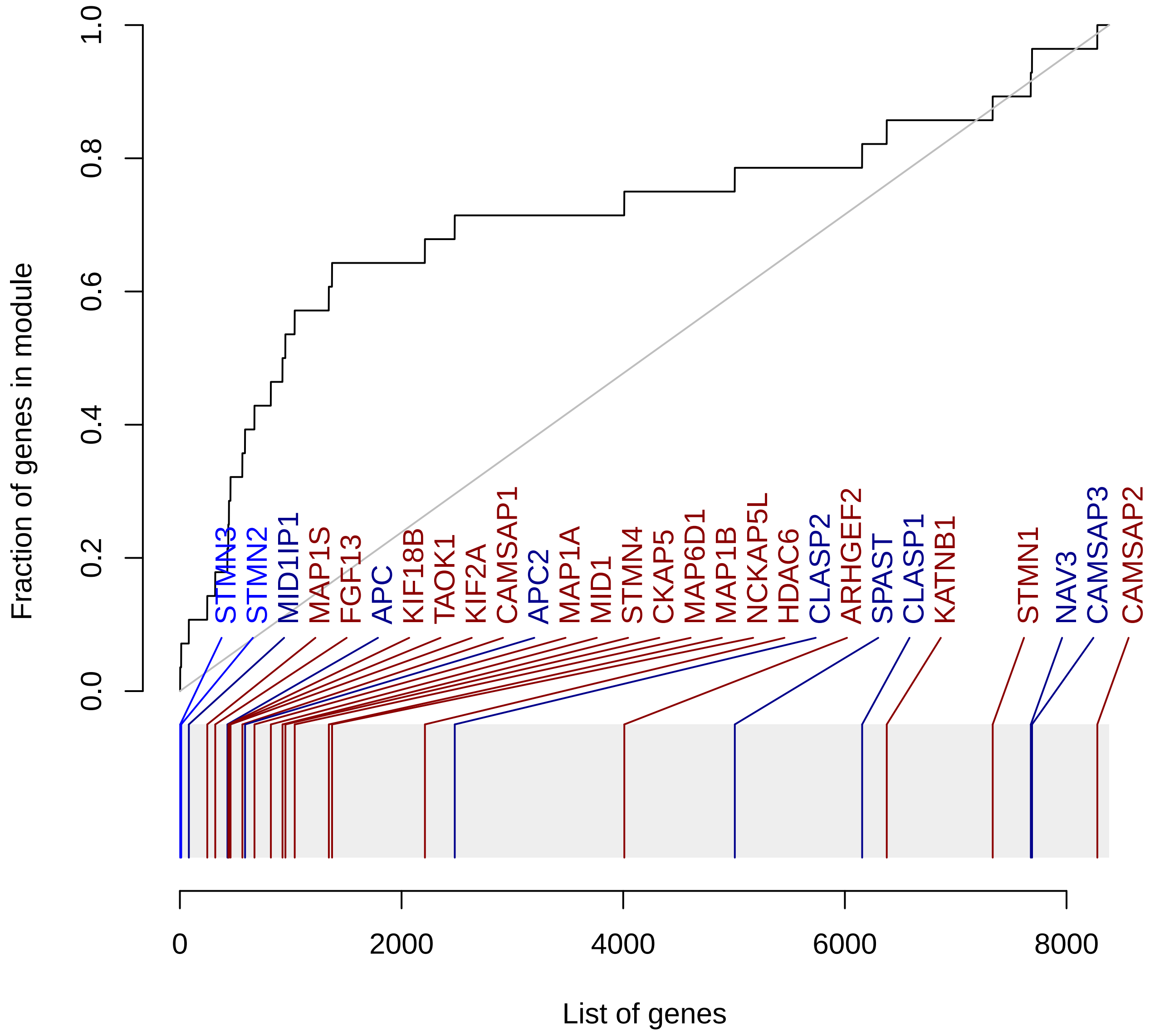
Fraction of genes in module
0.0
0.2
0.4
0.6
0.8
1.0
STMN3
STMN2
MID1IP1
MAP1S
FGF13
APC
KIF18B
TAOK1
KIF2A
CAMSAP1
APC2
MAP1A
MID1
STMN4
CKAP5
MAP6D1
MAP1B
NCKAP5L
HDAC6
CLASP2
ARHGEF2
SPAST
CLASP1
KATNB1
STMN1
NAV3
CAMSAP3
CAMSAP2
0
2000
4000
6000
8000
List of genes
